# Phase Response Design of Recursive All-Pass Digital Filters Using a Modified PSO Algorithm

**DOI:** 10.1155/2015/638068

**Published:** 2015-08-03

**Authors:** Wei-Der Chang

**Affiliations:** Department of Computer and Communication, Shu-Te University, Kaohsiung 824, Taiwan

## Abstract

This paper develops a new design scheme for the phase response of an all-pass recursive digital filter. A variant of particle swarm optimization (PSO) algorithm will be utilized for solving this kind of filter design problem. It is here called the modified PSO (MPSO) algorithm in which another adjusting factor is more introduced in the velocity updating formula of the algorithm in order to improve the searching ability. In the proposed method, all of the designed filter coefficients are firstly collected to be a parameter vector and this vector is regarded as a particle of the algorithm. The MPSO with a modified velocity formula will force all particles into moving toward the optimal or near optimal solution by minimizing some defined objective function of the optimization problem. To show the effectiveness of the proposed method, two different kinds of linear phase response design examples are illustrated and the general PSO algorithm is compared as well. The obtained results show that the MPSO is superior to the general PSO for the phase response design of digital recursive all-pass filter.

## 1. Introduction

An all-pass filter means that its magnitude response is exactly equal to some constant value at all frequencies and independent of frequencies. The function of the all-pass filter is mainly to offer a phase modification without changing the magnitude on a given filter. It is rather useful in the theory of minimum-phase systems, in transforming frequency-selective low-pass filters into other frequency-selective forms, and in obtaining variable-cutoff frequency-selective filters [1]. Due to these advantages, the all-pass filter has been applied in many signal processing applications, including the group-delay equalization, complementary filter banks, multirate filtering, and other fields [2–8]. A large number of methods for designing all-pass filter have been developed in recent years. In [2], for example, the author proposed a new design method for an all-pass filter where it has a least squares or an equiripple phase-error response. It is based on formulating a weighted error between the desired and the actual phase responses in a quadratic form. Filter coefficients can be solved by using a Toeplitz-plus-Hankel matrix. In [3], an IIR all-pass filter with equiripple phase response was designed based on the eigenvalue problem and this design problem can be formulated as the representation of an eigenvalue problem via the Remez exchange algorithm. A Hopfield neural network was combined to the design of IIR all-pass digital filters [5]. In the case, filter coefficients can be evaluated by Hopfield neural networks in a parallelism manner in accordance with the error function that is formulated as a Lyapunov energy function. In addition, the authors developed a digital linear phase notch filter design scheme based on IIR all-pass filter. The designed filter can be realized by parallel connection of two IIR all-pass filters with approximately linear phase. Design algorithms exhibit fast convergence and easy initial values determination [7].

Unlike the above-mentioned design schemes, this paper attempts to utilize a modified particle optimization (MPSO) algorithm to solve the digital recursive all-pass filter design problem. This developed algorithm is a variant of the general PSO but it has a better searching capacity in solving optimized problems. The detailed description for such MPSO algorithm will be addressed later. The remainder of this paper is summarized as follows.  Section 2 gives a brief description for the recursive all-pass digital filter. In Section 3, a modified PSO algorithm is introduced in detail and the MPSO-based design steps for all-pass digital filter are also given.  Section 4 will provide two different kinds of examples to confirm the applicability of the proposed method and some comparisons with the general PSO are further made. Finally, a conclusion about the proposed method is simply described in Section 5.

## 2. Recursive All-Pass Digital Filter

Let us consider a recursive all-pass digital filter whose transfer function is expressed by

(1) $$\begin{array}{c} H\left(z\right)=\frac{a_{N}+a_{N-1}z^{-1}+\cdots +a_{0}z^{-N}}{a_{0}+a_{1}z^{-1}+\cdots +a_{N}z^{-N}}=\frac{z^{-N}D\left(z^{-1}\right)}{D\left(z\right)}, \end{array}$$

where *N* represents the order of the filter, *a*_0_ is always set to be 1, *a*_1_, *a*_2_,…, *a*_*N*_ are real coefficients, and *D*(*z*) = ∑_*i*=0_^*N*^ *a*_*i*_ *z* ^−*i*^. Let *z* = *e* ^*jΩ*^, where *Ω* denotes the digital frequency, substituting it into (1) to derive the frequency response. The following magnitude response can be easily obtained:

(2) HΩ=z−NDz−1Dz=Dz−1Dz=1+a1ejΩ+⋯+aNejNΩ1+a1e−jΩ+⋯+aNe−jNΩ=1.

It is seen from (2) that the magnitude response is equal to one at all frequencies; that is, it is independent of the filter coefficients. Furthermore, its phase response is derived by

(3) θΩ=−NΩ+2arctan⁡∑i=1NaisiniΩ∑i=1NaicosiΩ=−NΩ+2arctan⁡asTΩ1+acTΩ,

where **s**(*Ω*) ≡ [sin(*Ω*), sin⁡(2*Ω*),…, sin⁡(*NΩ*)], **c**(*Ω*) ≡ [cos⁡(*Ω*), cos⁡(2*Ω*),…, cos⁡(*NΩ*)], and

(4) $$\begin{array}{c} a\equiv \left[a_{1},a_{2},\ldots ,a_{N}\right] \end{array}$$

is a parameter vector consisting of all filter coefficients. This vector fully dominates the phase response behavior of the digital filter. In this paper, we want to design the parameter vector **a** such that the phase response achieves certain design specification. Moreover, this vector **a** is called the particle or individual of the PSO algorithm and many such particles then form a population. Some adjusting mechanisms are utilized on the full population. Moreover, it can be easily seen from (3) that a highly complicated nonlinear function arctan⁡(·) is involved and it is difficult to solve. Thus, (3) always needs to be modified as another form for the phase response design [3–5]. However, the proposed method in this paper can directly use (3) for the phase response design of recursive all-pass filter.

## 3. Modified Particle Swarm Optimization (MPSO) Algorithm

Kennedy and Eberhart initially proposed the PSO algorithm in 1995 and recently it became one of the popular and efficient optimization algorithms [9]. Like most swarm intelligence algorithms, PSO is also a population-based search algorithm. It simulates the social behavior of organisms, such as fish schooling and bird flocking. Each fish or bird, viewed as a particle or an individual, represents a candidate solution to the optimized problem. By the velocity and position updating formulas, each particle moves through the search space toward the global solution. Based on the PSO algorithm, various engineering optimization applications have been successively developed and explored in recent years, such as power system stabilizer design [10], PID controller design [11, 12], FPGA implementations [13, 14], Volterra filter modeling [15], QRD-based multirelay system design [16], automatic clustering [17], multifault classification [18], and aeroengine nonlinear programming model [19]. Besides, the authors developed a novel PSO algorithm in which the inertia weight is modified to enhance its search capability [20]. The proposed method has successfully been applied in the high pass FIR digital filter design. Another design method for the low pass FIR digital filter with linear phase properties was also developed [21]. A new definition for the velocity vector and swarm updating of the PSO algorithm was proposed.

At the beginning, PSO algorithm requires an objective function to judge the performance of the particle and also to guide the search direction of the algorithm. To solve the phase response design problem for the recursive all-pass digital filter, the objective function (OF) is defined by

(5) $$\begin{array}{c} OF=\overset{\Omega_{max}}{\underset{\Omega_{min}}{\int }}\left|\theta_{d}\left(\Omega \right)-\theta \left(\Omega \right)\right|d\Omega , \end{array}$$

where *θ*_*d*_ is the desired phase response given by the designer, *θ* is the actual phase response of the all-pass digital filter as described by (3), and *Ω*_min_ and *Ω*_max_ are the integral lower and upper bounds, respectively. The algorithm is utilized to minimize this objective function OF to achieve the optimal phase response design. Each particle is changed according to the following velocity formula of (6) and position formula of (7) for original PSO algorithm:

(6) vijn+1=wvijn+c1r1pijn−aijn +c2r2gjn−aijn,

(7) $$\begin{array}{c} a_{ij}\left(n+1\right)=a_{ij}\left(n\right)+v_{ij}\left(n+1\right), \end{array}$$

where *n* denotes the *n*th iteration of the algorithm, *v*_*ij*_, *a*_*ij*_, and *p*_*ij*_ represent the velocity, position, and individual best position for the *i*th particle with respect to the *j*th dimension, respectively, *g*_*j*_ represents the global best position with respect to the *j*th dimension among the population, *w* is called the inertia weight, *c*_1_ and *c*_2_ are two positive acceleration coefficients that pull each particle toward the individual best and the global best positions, respectively, and *r*_1_ and *r*_2_ are two uniform distribution random numbers chosen from the interval [0,1]. The PSO algorithm uses these two updating mechanisms to achieve the optimization.

In this study, a modified PSO (MPSO) algorithm is taken into the phase response design of recursive all-pass digital filter [11, 15]. The difference between the original and modification is to change the velocity formula. In the MPSO, the population needs to be further divided into some subpopulations at the beginning; for example, suppose that the initial population includes 50 particles and it will be divided into five subpopulations. Thus, the first subpopulation is composed of particles from number one to number ten, and the second then contains particles from number eleven to number twenty, and so forth. The best particle of each subpopulation needs to be recorded according to its objective function. Instead of the velocity formula of (6), the MPSO algorithm uses the following improved version:

(8) vijn+1=wvijn+c1r1pijn−aijn +c2r2gjn−aijn+c3r3sjn−aijn,

where *s*_*j*_ is a new variable called the local best and represents the position of the best particle of the subpopulation where the *i*th particle is located, *c*_3_ is also a positive acceleration coefficient, and *r*_3_ is a random number selected from the range [0,1] uniformly.

Design steps of MPSO-based for the phase response design of the recursive all-pass digital filter can be summarized in the following.

*Data*. Filter order *N* in (1) and (3), desired phase response *θ*_*d*_ and integral lower and upper bounds *Ω*_min_ and *Ω*_max_ in (5), number of particles (population size) Ps, number of subpopulations *S*, number of generations *G*, inertia weight *w*, and positive constants *c*_1_, *c*_2_, and *c*_3_ in (8).

*Goal*. Derive a recursive all-pass digital filter with the phase response approaching the desired response *θ*_*d*_.

(1) Create an initial population consisting of Ps particles from the interval [−1,1] randomly.
(2) Divide the population into *S* subpopulations by particle serial numbers.
(3) If a prescribed number *G* of iterations are achieved, then the algorithm stops.
(4) Evaluate the objective function of (5) for each particle and record the related individual best, local best, and global best positions.
(5) Update each particle's velocity and position using (8) and (7).
(6) Go back to Step  (3).

## 4. Simulation Results

In this section, we consider two different examples with linear phase design to show the applicability of our proposed method [2, 5]. Some comparisons with the general PSO are also performed. In the PSO and MPSO, the variables of the algorithm are given by *w* = 0.8, *c*_1_ = *c*_2_ = 0.5, and *w* = 0.8, *c*_1_ = *c*_2_ = *c*_3_ = 0.5, respectively, for all of the following simulations.

**Example 1 .**

**Figure 1:**
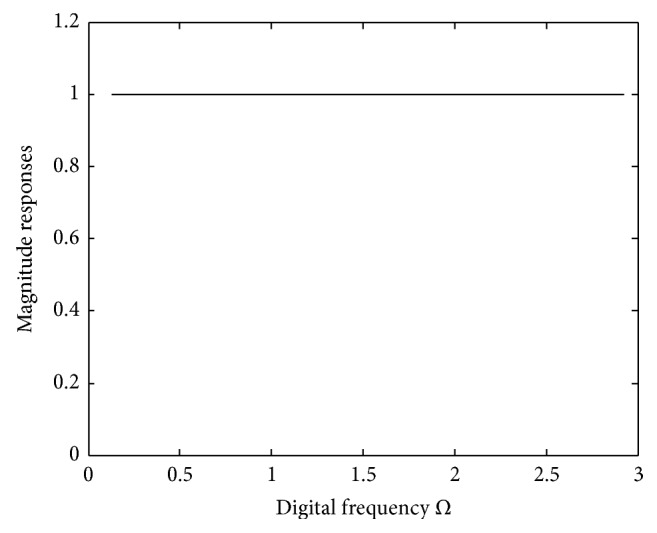
Magnitude response of Example 1.

**Figure 2:**
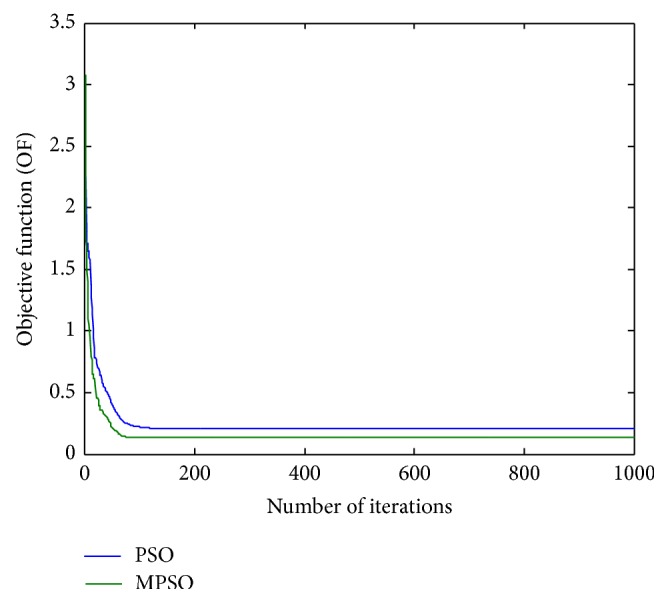
Trajectories of objective function (OF) for Run 1 of Example 1.

**Figure 3:**
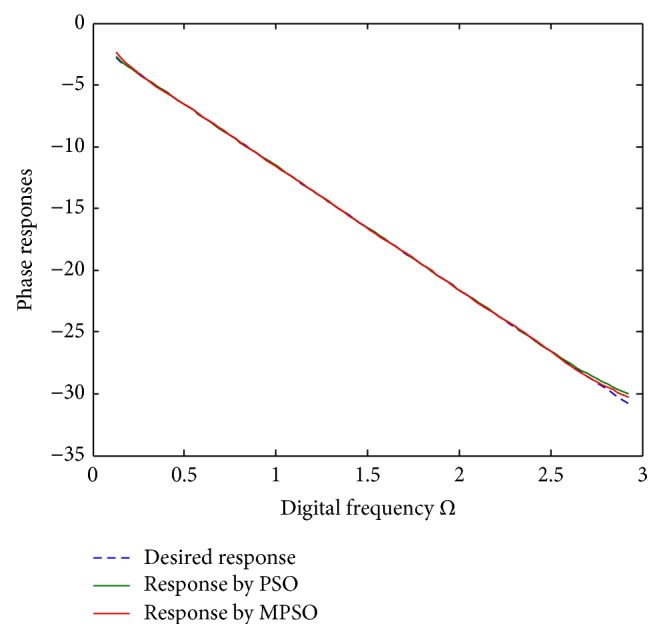
Phase responses for Run 1 of Example 1.

**Figure 4:**
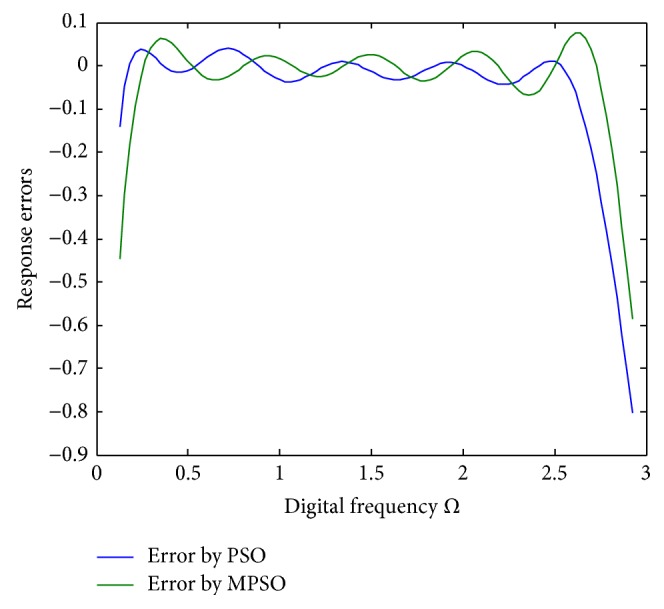
Phase response errors for Run 1 of Example 1.

In this example, the recursive all-pass filter is designed to approximate a desired Hilbert transformer whose phase response is given by

(9) $$\begin{array}{c} \theta_{d}\left(\Omega \right)=-N\Omega -\frac{\pi }{2},\;\Omega_{min}\leq \Omega \leq \Omega_{max}, \end{array}$$

where the lower bound and upper bound are set to *Ω*_min_ = 0.04*π* and *Ω*_max_ = 0.94*π*, respectively; *N* means the filter order and here it is chosen by *N* = 10. The magnitude response of such a recursive all-pass filter is plotted in Figure 1. In addition, the population size and number of generations are given by Ps = 20 and *G* = 1000 for the PSO and MPSO algorithm, and the number of subpopulations is simply set to *S* = 4 only for the MPSO. To verify the algorithm's robustness and efficiency, 20 independent runs with different initial conditions are executed for both algorithms. Final simulation results are listed in Tables 1 and 2 and shown in Figures 2–4, respectively.  Table 1 lists the objective function values of 20 independent runs and it clearly reveals that the results by the MPSO are better than those by the PSO for most of independent runs. The mean and variance are evaluated by *u* = 0.14269337 and *σ* ^2^ = 0.00035958 for the MPSO and *u* = 0.16607883 and *σ* ^2^ = 0.00313722 for the PSO, respectively. To show the design outcomes, Figure 2 displays the convergence trajectories of the objective function for Run 1 of the proposed MPSO and PSO algorithms. As can be seen from Figure 2, the MPSO algorithm has a quicker convergence and lower objective function value than the PSO algorithm. Both phase responses and errors are further shown in Figures 3 and 4, respectively. A better simulation result can be obtained by the proposed method. In addition, all of digital filter coefficients derived by Run 1 of the PSO and MPSO algorithm are listed in Table 2 for comparisons.

**Example 2 .**

**Figure 5:**
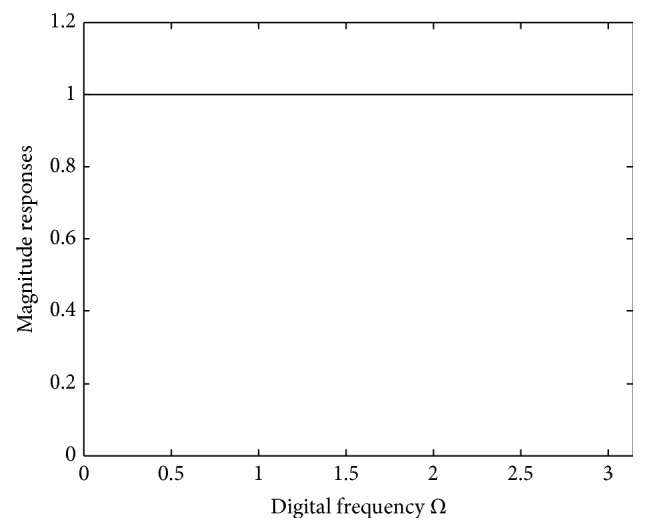
Magnitude response of Example 2.

**Figure 6:**
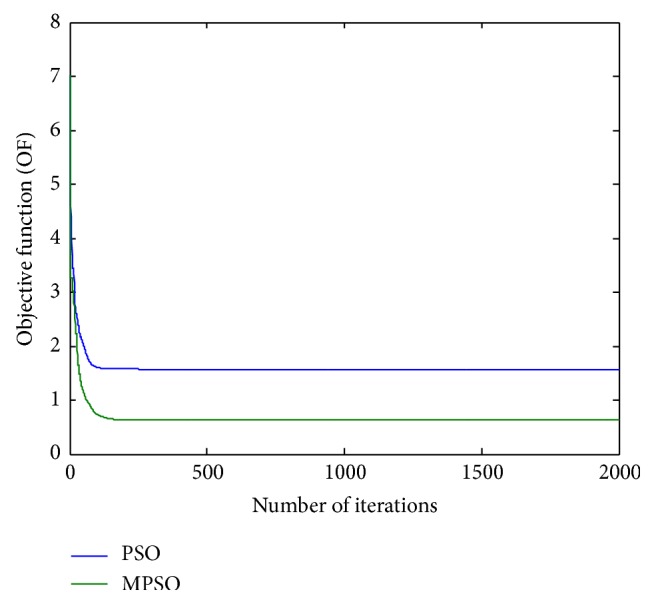
Trajectories of objective function (OF) for Run 1 of Example 2.

**Figure 7 fig7:**
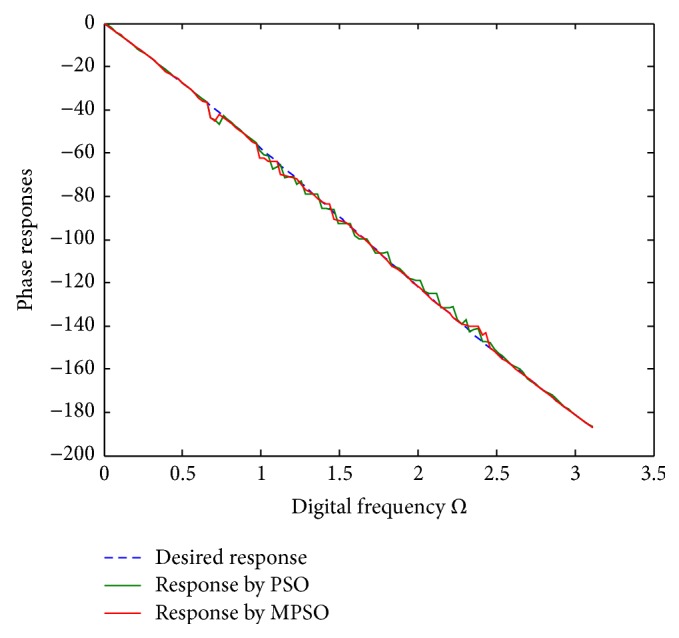
Phase responses for Run 1 of Example 2.

**Figure 8:**
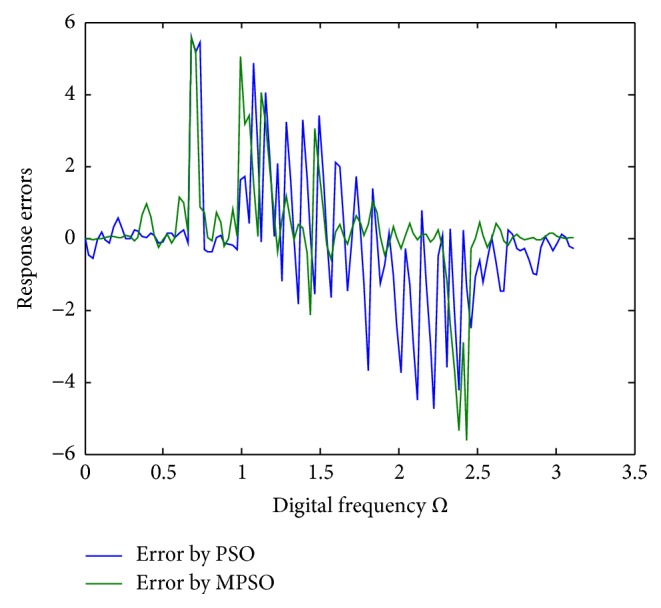
Phase response errors for Run 1 of Example 2.

This example will design a recursive all-pass digital filter with a desired sinusoidal phase response expressed by

(10) $$\begin{array}{c} \theta_{d}\left(\Omega \right)=4\pi \left(cos\Omega -1\right)-52\Omega ,\;\Omega_{min}\leq \Omega \leq \Omega_{max}, \end{array}$$

where *Ω*_min_ = 0 and *Ω*_max_ = *π* are given. Its corresponding magnitude response is shown in Figure 5. In the simulation, a digital recursive filter with *N* = 60 is adopted and the population size and iterative number of the algorithms are set to Ps = 40 and *G* = 2000, respectively, for solving such a higher-order digital filter. Moreover, as given in Example 1, the number of subpopulations is chosen by *S* = 4 and 20 independent runs with different sets of initial conditions are also performed for certifying the robustness of the algorithm.  Table 3 lists a comparison of the objective function values evaluated by the proposed MPSO and PSO for Run 1 to Run 20, respectively. Some of digital filter coefficients derived are listed in Table 4 for comparisons. Figures 6 [Fig fig7]–8 then show the related design outcomes only for Run 1 of the PSO and proposed algorithm. Again, it can be concluded from these results that the proposed MPSO is superior to the general PSO in the phase response design of recursive all-pass digital filter.

## 5. Conclusions

This paper has developed a new design method for the phase response design of recursive all-pass digital filter. A modified PSO (MPSO) algorithm is suggested to design the filter coefficients such that the obtained phase response can approximate the desired response that is given previously. The difference between the MPSO and PSO is to modify the velocity updating formula of the algorithm. To improve the search capacity, a new factor of local-best particle for each subpopulation is introduced in the modified velocity formula. Finally, two different kinds of examples have been illustrated to verify the efficiency of the proposed method as compared with the general PSO algorithm. Simulation results have sufficiently proven that the proposed MPSO has a better design outcome than the PSO in the phase response design of recursive all-pass digital filter.

## Figures and Tables

**Table 1:** Objective function values evaluated for Example 1.

	PSO algorithm	MPSO algorithm
Run 1	0.20570301	0.13606212
Run 2	0.13627085	0.13620043
Run 3	0.35740401	0.13679522
Run 4	0.13738652	0.13650124
Run 5	0.13672965	0.13680452
Run 6	0.14521057	0.13601797
Run 7	0.13619628	0.13673623
Run 8	0.15390549	0.13606745
Run 9	0.13755478	0.13762322
Run 10	0.14286368	0.13602069
Run 11	0.14405732	0.16636761
Run 12	0.14924150	0.13679615
Run 13	0.13622616	0.13703487
Run 14	0.13707200	0.13680303
Run 15	0.13634390	0.13701766
Run 16	0.21968176	0.22009637
Run 17	0.14488212	0.13649137
Run 18	0.14857613	0.13703097
Run 19	0.14250406	0.13624831
Run 20	0.27376677	0.14515192

Mean	0.16607883	0.14269337
Variance	0.00313722	0.00035958

**Table 2:** Digital filter coefficients derived by Run 1 of Example 1.

Filter coefficients	PSO algorithm	MPSO algorithm
*a*_1_	−0.974	−0.9853
*a*_2_	0.4350	0.4685
*a*_3_	−0.3959	−0.4388
*a*_4_	0.2323	0.2959
*a*_5_	−0.1912	−0.2625
*a*_6_	0.1048	0.1830
*a*_7_	−0.0743	−0.1485
*a*_8_	0.0268	0.0975
*a*_9_	−0.0153	−0.0646
*a*_10_	−0.0106	0.0290

**Table 3:** Objective function values evaluated for Example 2.

	PSO algorithm	MPSO algorithm
Run 1	1.56939914	0.62645705
Run 2	1.85475888	0.57827778
Run 3	1.55263227	0.67573844
Run 4	1.57989126	0.67840205
Run 5	2.03270089	0.75624957
Run 6	1.63204094	0.67220282
Run 7	1.34828024	0.52125315
Run 8	1.65465543	1.18349923
Run 9	1.39729362	0.86232224
Run 10	1.55541996	0.65710581
Run 11	1.82677089	1.14715629
Run 12	1.68802195	0.62355711
Run 13	1.94169732	0.65469452
Run 14	1.61249872	0.48998564
Run 15	1.78693672	1.17072011
Run 16	1.75210045	1.02655545
Run 17	1.57667970	0.76927394
Run 18	1.64655678	0.80753180
Run 19	1.84461988	1.10135973
Run 20	1.56655437	0.44814752

Mean	1.67097547	0.77252451
Variance	0.02816276	0.05174895

**Table 4:** Digital filter coefficients derived by Run 1 of Example 2.

Filter coefficients	PSO algorithm	MPSO algorithm
*a*_1_	0.0464	−0.1479
*a*_2_	0.8023	1.1317
*a*_3_	0.3584	0.0022
*a*_4_	0.8848	0.8390
*a*_5_	0.0292	−0.0636
*a*_6_	0.4525	0.5609
⋮	⋮	⋮
*a*_58_	0.0093	0.0089
*a*_59_	−0.1014	0.1059
*a*_60_	0.1847	0.0181

## References

[1] Oppenheim A. V., Schafer R. W., Buck J. R. (1999). *Discrete-Time Signal Processing*.

[2] Kidambi S. S. (1996). Weighted least-squares design of recursive allpass filters. *IEEE Transactions on Signal Processing*.

[3] Zhang X. (1999). Design of IIR digital allpass filters based on eigenvalue problem. *IEEE Transactions on Signal Processing*.

[4] Lang M. (1998). Allpass filter design and applications. *IEEE Transactions on Signal Processing*.

[5] Su L. C., Jou Y. D., Chen F. K., Sun C. M. Neural type least squares design for IIR all-pass filters.

[6] Horng J.-W. (2005). Current conveyors based allpass filters and quadrature oscillators employing grounded capacitors and resistors. *Computers and Electrical Engineering*.

[7] Stančić G., Nikolić S. (2013). Digital linear phase notch filter design based on IIR all-pass filter application. *Digital Signal Processing*.

[8] Chang W. D., Pan S. T., Cheng K. H., Hsu M. C. Optimal design of allpass digital filters using artificial bee colony.

[9] Kennedy J., Eberhart R. Particle swarm optimization.

[10] Abido M. A. (2002). Optimal design of power-system stabilizers using particle swarm optimization. *IEEE Transactions on Energy Conversion*.

[11] Chang W.-D., Shih S.-P. (2010). PID controller design of nonlinear systems using an improved particle swarm optimization approach. *Communications in Nonlinear Science and Numerical Simulation*.

[12] Chiou J.-S., Tsai S.-H., Liu M.-T. (2012). A PSO-based adaptive fuzzy PID-controllers. *Simulation Modelling Practice and Theory*.

[13] Cavuslu M. A., Karakuzu C., Karakaya F. (2012). Neural identification of dynamic systems on FPGA with improved PSO learning. *Applied Soft Computing Journal*.

[14] Vasumathi B., Moorthi S. (2012). Implementation of hybrid ANNPSO algorithm on FPGA for harmonic estimation. *Engineering Applications of Artificial Intelligence*.

[15] Chang W.-D. (2012). Volterra filter modeling of nonlinear discrete-time system using improved particle swarm optimization. *Digital Signal Processing*.

[16] Zhang C., Wu M., Luan L. (2013). An optimal PSO distributed precoding algorithm in QRD-based multi-relay system. *Future Generation Computer Systems*.

[17] Nanda S. J., Panda G. (2013). Automatic clustering algorithm based on multi-objective Immunized PSO to classify actions of 3D human models. *Engineering Applications of Artificial Intelligence*.

[18] Liu Z., Cao H., Chen X., He Z., Shen Z. (2013). Multi-fault classification based on wavelet SVM with PSO algorithm to analyze vibration signals from rolling element bearings. *Neurocomputing*.

[19] Wang Y., Li B., Sun T., Jiang K., Wang X. A modified PSO algorithm for aero-engine non-linear programming model.

[20] Mondal S., Chakraborty D., Kar R., Mandal D., Ghoshal S. P. Novel particle swarm optimization for high pass FIR filter design.

[21] Mukherjee S., Kar R., Mandal D., Mondal S., Ghoshal S. P. Linear phase low pass FIR filter design using improved particle swarm optimization.

